# Goals of internet use and subjective safety of adolescents on the internet

**DOI:** 10.1192/j.eurpsy.2021.588

**Published:** 2021-08-13

**Authors:** L. Shaigerova, R. Shilko, O. Almazova

**Affiliations:** Faculty Of Psychology, Lomonosov Moscow State University, Moscow, Russian Federation

**Keywords:** goals of Internet use, adolescents, subjective safety

## Abstract

**Introduction:**

Modern teenagers spend most of their lives on social networks and the Internet, meeting various needs. At the same time, more detailed research is needed on how specific Internet use affects various aspects of the psychological state.

**Objectives:**

The objective is to identify how the main goals of Internet use by adolescents are related to their subjective safety on the Internet and self-assessment of health.

**Methods:**

The study involved 480 participants from 15 to 18 years old. We analyzed the main reasons for respondents’ use of the Internet (7 main goals were highlighted) and uncovered the relationship between the main goals of Internet use, self-assessment of health and subjective safety on the Internet.

**Results:**

Adolescents who identified communication (t = -2.450, p=0.015) and shopping and receiving services as their main goals for using the Internet rated their health as significantly worse (t = -3.170, p = 0.002). Young men who use the Internet more often as a source of information feel significantly less secure on the Internet (t = -2.237, p=0.026), as do those who use the Internet more often to expand communication in social networks (t = -2.040, p=0.043). For girls, the goals of using the Internet and the sense of subjective security on the Internet were not significantly related.

**Conclusions:**

Using the Internet to communicate, buy and receive services, and search for information can negatively affect the assessment of own health and subjective safety, especially among young people. The research was supported by the Russian Science Foundation, with the grant 15-18-00109.

